# Developing a strategic plan for a climate-resilient health system in Iran

**DOI:** 10.1371/journal.pgph.0006836

**Published:** 2026-08-07

**Authors:** Ali Mohammad Mosadeghrad, Mahnaz Afshari, Hamed Dehnavi, Iman Keliddar, Maryam Zahmatkesh, Parvaneh Isfahani, Tahere Sharifi, Abbas Shahsavani, Abbas Ostadtaghizadeh, Masoumeh Abbasabadi-Arab, Masud Yunesian

**Affiliations:** 1 School of Public Health, Climate Change and Health Research Centre, Tehran University of Medical Sciences, Tehran, Iran; 2 Social Determinants of Health Research Center, Saveh University of Medical Sciences, Saveh, Iran; 3 School of Public Health and safety, Shahid Beheshti University of Medical Sciences, Tehran, Iran; 4 School of Public Health, Ahvaz Jundishapur University of Medical Sciences, Ahvaz, Iran; 5 School of Business and Management, Royal Holloway University of London, London, United Kingdom; 6 School of Public Health, Zabol University of Medical Sciences, Zabol, Iran; 7 Nursing Care Research Center, Semnan University of Medical Sciences, Semnan, Iran; 8 Department of Environmental Health Engineering, School of Public Health and Safety, Shahid Beheshti University of Medical Sciences, Tehran, Iran; 9 School of Public Health, Climate change and health research Centre, Tehran University of Medical Sciences, Tehran, Iran; 10 Prehospital Emergency Research Center, National Emergency Medical Organization, Tehran. Iran; 11 School of Public Health, Tehran University of Medical Sciences, Tehran, Iran; Sustainable Futures Collaborative, INDIA

## Abstract

Climate change is a long-standing natural process that has been significantly accelerated in recent decades by anthropogenic activities, resulting in profound shifts in climate patterns and escalating risks to human health. Iran, characterized by predominantly arid and semi-arid conditions, chronic water scarcity, and complex socio-economic constraints, is highly vulnerable to climate-related hazards, including droughts, floods, heatwaves, air pollution, and climate-sensitive diseases. In this context, health systems have a dual responsibility: to strengthen adaptive capacity and resilience while simultaneously reducing their own carbon footprint. This study aimed to develop the National Strategic Plan for a Climate-Resilient and Environmentally Sustainable Health System in Iran (2023–2027) using a participatory action research approach. A multi-sectoral national committee conducted a Strengths, Weaknesses, Opportunities, and Threats analysis to assess internal and external determinants of climate resilience within Iran’s health system. Quantitative scoring of strategic factors indicated a mean score of 2.47/4 for internal factors and 2.12/4 for external factors, positioning the health system within a precautionary strategic quadrant, characterized by limited internal capacity and substantial external threats. Based on these findings, a strategic plan was formulated, including a clearly defined vision, mission, values, seven strategic goals, twenty objectives, and a set of 181 prioritized actions. The findings demonstrate that while Iran’s health system possesses important structural strengths, particularly in primary health care, it faces critical challenges related to financing, workforce preparedness, and environmental pressures. This study presents a set of strategies and actions to strengthen the climate resilience of Iran’s health system by 2027, emphasizing governance, financing, workforce capacity, infrastructure, information systems, and service delivery. This strategic plan provides an evidence-informed and context-specific roadmap to guide policy-makers and health managers in strengthening climate resilience and environmental sustainability, with potential relevance for other middle-income countries confronting similar climate vulnerabilities.

## Introduction

Iran is among the countries most vulnerable to the impacts of climate change due to its predominantly arid and semi-arid climate, limited water resources, high evaporation rates, and increasing exposure to extreme weather events. More than 80% of Iran’s land area is classified as arid or semi-arid, with average annual precipitation less than one-third of the global mean [1]. Climate projections indicate rising temperatures, prolonged droughts, more intense floods, and increasingly frequent dust storms, all of which pose significant risks to population health and place growing pressure on health systems [2].

Evidence from Iran demonstrates that climate change is already affecting health outcomes through multiple direct and indirect pathways. Heatwaves have been associated with increased cardiovascular and respiratory morbidity and mortality, while worsening air pollution- particularly in large urban areas- has intensified the burden of non-communicable diseases [3]. Climate variability has also altered the distribution and seasonality of climate-sensitive infectious diseases, including vector-borne and waterborne illnesses [4,5]. In addition, droughts and environmental degradation have negatively affected food security and nutritional status, increasing vulnerability among children and other at-risk populations [6]. Beyond physical health impacts, climate-related disasters and environmental stressors have contributed to mental health challenges, displacement, and social disruption [7].

Iran’s health system, while relatively strong in primary health care (PHC) coverage and service delivery, is increasingly exposed to climate-related risks that threaten its stability and performance. Climate hazards can damage health infrastructure, disrupt supply chains for medicines and medical equipment, and overwhelm health facilities during extreme events [8]. At the same time, structural challenges-including limited sustainable financing, shortages of specialized human resources in climate and environmental health, and fragmented institutional arrangements-constrain the system’s adaptive capacity [9]. Community-based health workers are a key component of Iran’s PHC system, particularly in rural and underserved areas, where they serve as frontline providers of health promotion, disease prevention, surveillance, and emergency response. However, they are also directly exposed to climate-related hazards, including extreme heat, air pollution, floods, and climate-sensitive infectious disease outbreaks, which can negatively affect their health, safety, and service delivery capacity. Despite these risks, they play an important role in enhancing community adaptation by supporting risk communication, facilitating early warning dissemination, and strengthening climate-sensitive disease surveillance at the local level. Therefore, strengthening the capacity, occupational safety, and preparedness of community-based health workers is essential for building a climate-resilient health system, particularly in low- and middle-income countries facing increasing climate variability and extreme events [10]. Without deliberate and coordinated action, climate change risks undermining past gains in population health, equity, and health system performance.

Recognizing these challenges, the World Health Organization (WHO) has developed an operational framework for building climate-resilient health systems, emphasizing six core health system building blocks: governance and leadership, financing, health workforce, health information systems, infrastructure and technologies, and service delivery [11]. This framework underscores the importance of systematically integrating climate considerations into health system planning and decision-making, supported by multi-sectoral collaboration, systems thinking, and evidence-informed strategies. In recent years, global evidence has increasingly highlighted that health systems must not only adapt to climate change but also contribute to mitigation efforts, as the health sector itself accounts for approximately 5% of global greenhouse gas emissions [12].

**Fig 1:**
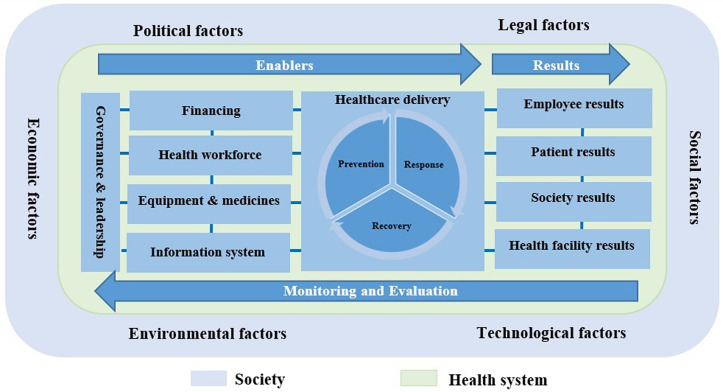
A conceptual model of a climate-resilient health system.

Fig 1 illustrates the framework of a climate-resilient health system [9]. Achieving sustainable outcomes for health facilities, personnel, patients, and the wider community requires systematic strengthening of the system’s core functions- governance, financing, workforce, infrastructure, health information systems, and service delivery. When these foundations are robust, health facilities are better equipped to withstand environmental stressors, healthcare providers benefit from improved job security, psychological support, and professional preparedness, and patients receive safer and more equitable care. At the community level, this translates into enhanced health outcomes, greater social stability, and stronger readiness for climate-related challenges. Employing a systems-thinking approach, which acknowledges feedback loops, interdependencies, and the inherent complexity of health systems, is essential for designing adaptive policies and interventions. Building resilience therefore extends beyond technical measures; it requires institutional transformation, cross-sectoral collaboration, inclusive governance, and sustained investment in adaptive capacity.

Despite growing recognition of climate-related health risks in Iran, there has been no comprehensive, nationally coordinated strategic plan explicitly designed to strengthen climate resilience and environmental sustainability across all functions of the health system. Existing initiatives are often fragmented, short-term, or insufficiently aligned with health system building blocks and long-term development priorities [13]. This gap highlights the need for a structured, participatory, and systems-oriented approach to strategic planning that reflects national priorities, institutional capacities, and external constraints, including economic pressures and international sanctions.

Accordingly, the aim of this study was to develop a national strategic plan to enhance climate resilience and environmental sustainability within Iran’s health system using a participatory action research approach. Guided by the WHO operational framework for climate-resilient health systems, the study sought to systematically assess internal and external determinants of resilience, identify strategic priorities, and formulate context-specific goals, objectives, and actions. By documenting both the process and outcomes of this strategic planning exercise, this study aims to provide an evidence-informed roadmap for policymakers and health system managers in Iran and to contribute to the global literature on strengthening climate-resilient health systems in middle-income countries.

## Methods

### Study design and methodological approach

This study adopted a Participatory Action Research (PAR) design to develop a national strategic and operational plan for strengthening climate resilience and environmental sustainability within Iran’s health system. PAR is a well-established, rigorous research methodology that emphasizes collaborative knowledge production through iterative cycles of problem identification, planning, action, reflection, and evaluation, actively engaging stakeholders who are directly involved in or affected by the issue under investigation [14,15]. In the context of complex system-level challenges such as climate change and health system resilience, PAR is particularly appropriate, as it integrates empirical evidence with experiential knowledge and supports the co-creation of context-specific, actionable solutions.

The epistemological stance of this study was pragmatic and systems-oriented, recognizing health systems as complex adaptive systems shaped by dynamic interactions between institutional, environmental, social, and political factors [16]. Within this framework, PAR served as the overarching methodological approach, while specific qualitative and quantitative techniques were employed as complementary tools to inform different phases of the participatory process.

### Study setting and duration

The study was conducted at the national level in Iran between January and March 2023, under the stewardship of the Center for Environmental and Occupational Health of the Ministry of Health and Medical Education. The research focused on the development of a five-year national strategic plan (2023–2027) and a corresponding one-year operational plan, aimed at enhancing climate resilience across core health system functions.

### Participatory structure and stakeholder engagement

A national strategic planning committee was established as the central participatory body guiding the research process. The committee comprised policymakers, senior managers, technical experts, and academic specialists from the Ministry of Health, medical universities, and relevant research centers. In addition, representatives from environmental health departments and disaster management authorities participated in selected sessions. Participants were purposively selected based on their roles, expertise, and decision-making authority in health system governance, climate change, environmental health, disaster risk management, and health services delivery.

Stakeholder engagement followed core PAR principles, including shared decision-making, collective reflection, and iterative feedback. Committee members were actively involved in defining the research problem, interpreting findings, prioritizing strategic issues, and co-developing the strategic and operational plans.

### PAR cycles and research phases

**Fig 2:**
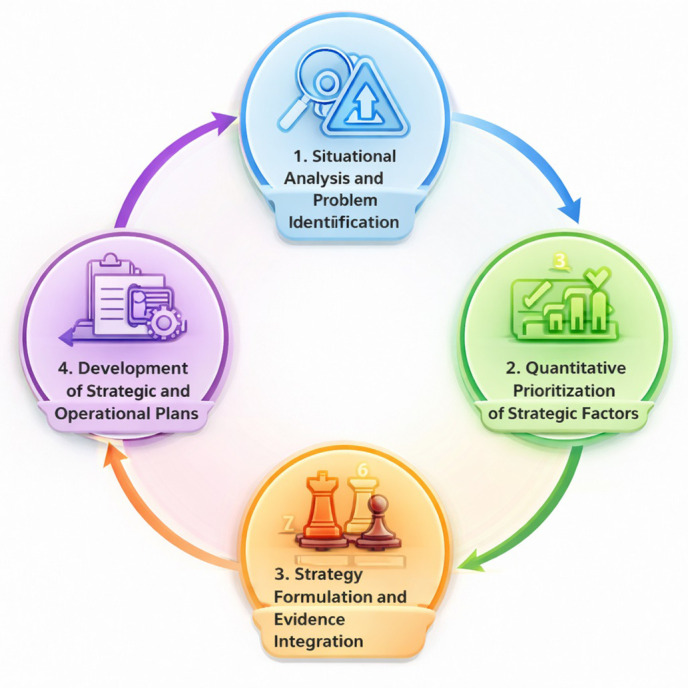
PAR cycle for developing a climate-resilient health system strategic plan in Iran.

This study was guided by the World Health Organization’s operational framework for climate-resilient health systems [11] and aligned with established PAR frameworks in health systems research [14]. A schematic representation of the PAR cycle used in this study is presented in Fig 2. The cycle consisted of iterative phases of (1) situational analysis and problem identification, (2) quantitative prioritization of strategic factors, (3) strategy formulation and evidence integration, and (4) development of strategic and operational plans. Continuous stakeholder engagement, collective reflection, and feedback informed all phases of the cycle.

#### Phase 1: Situational Analysis and Problem Identification.

In the first phase, participatory brainstorming sessions were conducted to identify key internal and external factors influencing the climate resilience of Iran’s health system. These discussions were guided by the World Health Organization’s operational framework for climate-resilient health systems, ensuring alignment with internationally recognized health system building blocks [11]. A Strengths, Weaknesses, Opportunities, and Threats (SWOT) analysis was employed to identify internal and external determinants of climate resilience within Iran’s health system. In this study, SWOT functioned as an analytic instrument within the PAR framework rather than as an independent methodology.

#### Phase 2: Quantitative Prioritization of Strategic Factors.

Based on the outputs of the brainstorming sessions, a structured questionnaire was developed to quantify the relative importance of identified SWOT factors. Committee members independently rated each item using a four-point Likert scale (from 1 = strongly disagree to 4 = strongly agree). Mean scores and standard deviations were calculated for internal (strengths and weaknesses) and external (opportunities and threats) factors. These quantitative results were used to construct a strategic positioning matrix, which enabled the classification of the health system’s overall strategic position with respect to climate resilience. This step enhanced methodological rigor by complementing qualitative insights with quantitative prioritization and transparency.

#### Phase 3: Strategy Formulation and Evidence Integration.

In the third phase, the strategic direction of the climate-resilient health system plan was collaboratively defined. This included the formulation of the mission, vision, core values, strategic goals, and objectives, guided by the Mosadeghrad Strategic Planning Model [17]. To ensure that proposed strategies were evidence-informed, a mixed qualitative evidence synthesis approach was embedded within the PAR cycle. This involved:

1. A scoping review of international and national literature on climate-resilient health systems, adaptation strategies, and health system strengthening [18].
2. Semi-structured interviews with national experts in climate change, environmental health, health policy, and disaster management to capture contextual insights and feasibility considerations [9].
3. Additional participatory group sessions to integrate empirical evidence with stakeholder perspectives and prioritize feasible interventions.

These methods were deliberately sequenced to inform collective reflection and decision-making within the PAR process.

#### Phase 4: Development of Strategic Plan.

In the final phase, the committee translated strategic priorities into a national strategic plan (2023–2027) and a detailed operational plan, specifying goals, objectives, actions, responsible units, and implementation timelines. Emphasis was placed on aligning proposed actions with existing institutional capacities, resource constraints, and governance structures within Iran’s health system.

Throughout this phase, iterative feedback loops were used to refine the plans, ensuring coherence across health system building blocks and consistency with national policies and international frameworks.

### Ethical considerations

Ethical approval for the study was obtained from the Ethics Committee of Tehran University of Medical Sciences (Approval Code: IR.TUMS.SPH.REC.1401.098). All participants were informed about the study objectives, procedures, and their voluntary role in the research process. Verbal informed consent was obtained prior to participation, and confidentiality of individual contributions was maintained throughout all phases of the study.

## Results

### Participatory situational analysis

#### Internal factors: Strengths and weaknesses.

The participatory situational analysis identified several internal strengths that support climate resilience within Iran’s health system. Key strengths included a well-established primary health care (PHC) network, effective collaboration between health centers, communities, and local authorities, and the presence of specialized research centers in health, environment, and climate-related fields. Participants also emphasized the role of existing hospital safety accreditation programs, communication channels with international organizations, and the availability of experienced scientific and technical experts across multiple disciplines as enabling factors for climate-resilient health system development.

In contrast, major internal weaknesses were consistently highlighted across PAR sessions. These included limited institutional commitment from senior leadership, the absence of a dedicated organizational unit or focal point for climate and health at medical universities, and shortages of specialized human resources with expertise in climate-resilient health system planning. Structural constraints such as unsustainable financing mechanisms, low climate resilience of health facilities, insufficient safety standards in infrastructure, weaknesses in supply chain management, and periodic shortages of essential medicines and equipment were also identified as critical barriers to effective adaptation.

#### External factors: Opportunities and threats.

With respect to external opportunities, participants identified Iran’s membership in international climate agreements, including the United Nations Framework Convention on Climate Change, as an enabling context for inter-sectoral collaboration. Additional opportunities included the existence of a National Climate Change Office within the Department of Environment, a national crisis management organization, and formal inter-sectoral coordination mechanisms such as the Supreme Council for Health and Food Security. Participants also noted that existing regulatory frameworks, including the Clean Air Act and engineering and construction standards, provide a policy foundation for advancing climate-resilient health infrastructure.

Conversely, the primary external threats to health system climate resilience were perceived to stem from intensifying climate variability, rising temperatures, and changing disease patterns. Iran’s relatively high greenhouse gas emissions and continued dependence on fossil fuels were identified as structural threats that exacerbate environmental degradation. Participants further emphasized that economic and political sanctions restrict access to international financing mechanisms, advanced technologies, and global knowledge networks, thereby limiting opportunities for large-scale infrastructure adaptation and innovation. Climate-induced pressures on food security and agricultural productivity were also recognized as indirect but significant threats to population health and health system stability.

### Quantitative assessment of internal and external factors

The quantitative prioritization of SWOT factors provided further insight into the relative strengths and vulnerabilities of the health system. The mean score for internal factors (strengths and weaknesses) was 2.47 out of 4, indicating moderate internal capacity for climate resilience. Among internal dimensions, governance and leadership received the highest mean score (2.67), followed by service delivery (2.58), suggesting relative strengths in organizational structure and frontline health services. In contrast, financing (2.03) and the health workforce (2.28) received the lowest scores, highlighting critical areas requiring targeted investment and capacity building.

The mean score for external factors (opportunities and threats) was 2.12 out of 4, reflecting a challenging external environment. Legal factors achieved the highest score (2.50), indicating favorable policy opportunities, while economic (1.49) and environmental (1.67) factors were rated lowest, underscoring substantial macroeconomic and ecological constraints. Detailed scores for each dimension are presented in Table 1.

**Table 1:** Mean scores of determinants of climate resilience of Iran’s health system (out of 4).

Internal factors	Mean	SD	External factors	Mean	SD
Governance and leadership	2.67	0.44	Political factors	2.23	0.55
Financing	2.03	0.15	Economic factors	1.49	0.19
Health workforce	2.28	0.32	Social factors	2.16	0.27
Facilities, equipment & medicines	2.52	0.32	Technological factors	2.37	0.29
Health information system	2.36	0.29	Environmental factors	1.67	0.28
Health service delivery	2.58	0.31	Legal factors	2.50	0.37
Key performance results	2.34	0.26			
Score of internal factors	2.47	0.37	Score of external factors	2.12	0.48

### Strategic positioning of the health system

**Fig 3:**
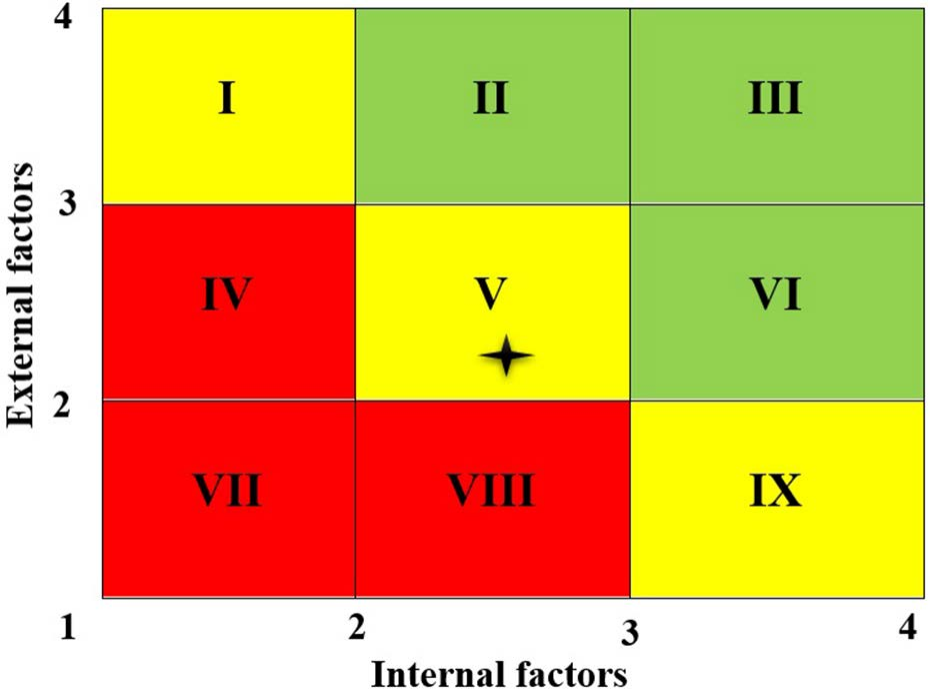
Strategic position matrix of Iran’s health system resilience to climate change.

The scores obtained for internal and external factors in the Strategic Position Matrix will indicate the strategic position of Iran’s health system’s climate resilience (Fig 3). Colour coding corresponds to quadrant classification based on the SWOT analysis: green denotes an aggressive/developmental posture, yellow denotes a precautionary/defensive posture, and red denotes a retreat/retrenchment posture.

Using the aggregated internal and external scores, the strategic positioning matrix classified Iran’s health system within a precautionary strategic quadrant (Fig 2). This position reflects a combination of moderate internal capacity and pronounced external threats, suggesting that the health system should prioritize strategies aimed at mitigating vulnerabilities while preserving and strengthening existing capacities. Based on this positioning, participants collectively identified precautionary strategies, including strengthening the climate resilience of health infrastructure, improving resource efficiency, and cost management, enhancing emergency preparedness and response capacity, expanding internal and external partnerships to compensate for structural and financial constraints; and optimizing health service delivery through quality improvement to enhance the system’s overall resilience.

### Strategic outputs of the participatory action research process

The final outputs of the PAR process included the articulation of a shared mission, vision, and core values for a climate-resilient and environmentally sustainable health system. The mission emphasized safeguarding population health through a climate-adaptive and resilient health system, while the vision aimed to position Iran’s health system among the leading climate-resilient systems in the WHO Eastern Mediterranean Region by 2027. Core values encompass intra- and inter-sectoral collaboration, teamwork, equity, accountability, transparency, customer orientation, and health-centeredness.

Seven strategic goals were formulated, aligned with the WHO health system building blocks: enhancing governance and leadership; ensuring sustainable financing; developing a competent climate-resilient health workforce; enhancing infrastructure, equipment, and supplies; expanding climate-adapted health information systems; strengthening health service delivery; and improving overall health system performance. These goals were operationalized through 20 objectives and 181 actions, which collectively defined the system’s implementation priorities (Table 2).

**Table 2:** Goals, Objectives, and Actions of Iran’s health system climate resilient strategic plan.

Goals	Objectives	Actions
G1 - Strengthening governance of the climate-resilient health system	3	33
G2 - Ensuring sustainable financing for the climate-resilient health system	2	7
G3 - Developing the health workforce of the climate-resilient health system	3	18
G4 - Enhancing infrastructure, and supplies of the climate-resilient health system	3	28
G5 - Strengthening the health information of the climate-resilient health system	3	36
G6 - Strengthening the service delivery of the climate-resilient health system	3	40
G7 - Improving the performance of the climate-resilient health system	3	19
**7**	**20**	**181**

This strategic plan offers a detailed roadmap aimed at strengthening the national health system’s ability to safeguard public health in the context of climate variability. The participatory process produced a structured and coherent strategic framework that clarifies institutional roles, priority areas for investment, and mechanisms for monitoring progress, thereby providing a foundation for phased implementation and adaptive learning in response to evolving climate risks. Although the primary focus is on the Ministry of Health and its affiliated institutions, successful implementation necessitates coordinated efforts with other ministries and government agencies.

## Discussion

This study developed a national strategic and operational plan to strengthen climate resilience and environmental sustainability within Iran’s health system using a participatory action research approach. The findings indicate that while Iran’s health system possesses important structural strengths- particularly in primary health care coverage, governance mechanisms, and technical expertise- it faces substantial internal and external constraints that limit its adaptive capacity. The strategic positioning of the health system within a precautionary quadrant reflects the need for deliberate, phased, and risk-informed strategies that prioritize vulnerability reduction while consolidating existing capacities.

One of the most significant findings of this study is the relatively stronger performance of governance and service delivery compared with other health system building blocks. Iran’s PHC network provides a critical foundation for climate resilience, enabling early detection of climate-sensitive diseases, community-level surveillance, and equitable access to essential services during climate-related shocks. This aligns with international evidence highlighting PHC as a cornerstone of climate-resilient health systems, particularly in low- and middle-income countries where community-based platforms play a central role in adaptation and emergency response [19,20]. However, the effectiveness of PHC in addressing climate risks depends on its integration with climate information systems, early warning mechanisms, and multi-sectoral coordination, which remain underdeveloped in Iran.

In contrast, financing and health workforce capacity emerged as the weakest internal dimensions of climate resilience. Insufficient and unstable funding constrains investments in climate-resilient infrastructure, preparedness planning, and workforce development. Similar challenges have been reported in other middle-income settings, where climate adaptation in the health sector often relies on short-term project-based funding rather than sustainable financing mechanisms [17,21]. The shortage of specialized human resources with expertise in climate change, environmental health, and disaster risk management further undermines adaptive capacity. Strengthening climate-related competencies through pre-service education, in-service training, and dedicated career pathways is therefore essential to ensure that health systems can anticipate and respond effectively to evolving climate risks [17,22].

The external environment presents both enabling conditions and significant threats. Favourable legal and policy frameworks, including Iran’s participation in international climate agreements and the existence of inter-sectoral coordination bodies, provide opportunities to mainstream health considerations into national climate policies. The WHO operational framework for climate-resilient health systems emphasizes that such policy coherence and multi-sectoral governance are critical enablers of effective adaptation [11]. High-level inter-sectoral mechanisms can successfully integrate health priorities into national adaptation agendas, improving policy alignment and resource mobilization.

Nevertheless, the findings also highlight substantial external threats, particularly those related to economic constraints, environmental degradation, and political factors. Economic pressures and international sanctions limit access to global climate financing mechanisms, advanced technologies, and international knowledge networks, thereby constraining large-scale investments in resilient health infrastructure and low-carbon technologies. In addition, Iran’s continued reliance on fossil fuels and exposure to compound climate hazards, including droughts, heatwaves, dust storms, and food insecurity, create systemic risks that extend beyond the mandate of the health sector alone [18]. Moreover, ongoing geopolitical tensions and regional conflicts in the broader Middle East context, including persistent instability and security pressures involving Iran and other regional and global actors, may further weaken health system resilience by disrupting service delivery, damaging infrastructure, constraining supply chains for essential medicines and equipment, and limiting investment in long-term health system strengthening. These conditions can also intensify workforce challenges and exacerbate inequities in access to care during crises. These interconnected threats underscore the importance of adopting holistic approaches, such as One Health and Planetary Health frameworks, which recognize the interdependence of human, animal, and ecosystem health.

The precautionary strategic position identified in this study suggests that Iran’s health system should adopt defensive and adaptive strategies focused on safeguarding core functions while incrementally building resilience. Rather than pursuing ambitious but potentially unfeasible reforms, the findings support a phased approach that emphasizes strengthening governance structures, improving resource efficiency, enhancing emergency preparedness, and fostering strategic partnerships. This approach is consistent with the adaptive pathways framework promoted by the Intergovernmental Panel on Climate Change, which advocates iterative planning, continuous learning, and flexible decision-making under conditions of uncertainty [23].

Importantly, this study reinforces the growing global consensus that climate-resilient health systems must address both adaptation and mitigation objectives. Health systems contribute an estimated 5% of global greenhouse gas emissions, and failure to reduce this footprint risks undermining broader climate goals [12]. Integrating low-carbon considerations into health infrastructure design, procurement practices, and service delivery is therefore not only an environmental imperative but also a moral and strategic responsibility. For Iran, incremental investments in energy-efficient facilities, renewable energy sources, and green supply chains could simultaneously enhance resilience, reduce operational costs, and improve service continuity during climate-related disruptions.

The strategic framework developed through this participatory process provides a practical roadmap for implementation within Iran’s institutional and resource constraints. The seven strategic goals and associated objectives align closely with the WHO health system building blocks, facilitating integration into existing planning, budgeting, and monitoring mechanisms. However, successful implementation will depend on sustained political commitment, clear institutional accountability, and effective inter-sectoral collaboration. International development partners, including UN agencies and global health initiatives, may also play a supportive role by facilitating technical assistance, capacity building, and access to global knowledge resources, particularly in areas constrained by domestic limitations.

This study has several implications for policy and practice. First, climate resilience should be institutionalized as a core function of health system governance rather than treated as a peripheral or project-based activity. Second, investments in workforce development and sustainable financing are critical leverage points for strengthening adaptive capacity. Third, inter-sectoral coordination mechanisms must be strengthened to address upstream environmental and social determinants of climate-related health risks. Finally, continuous monitoring, evaluation, and learning mechanisms are essential to ensure that strategies remain responsive to evolving climate conditions and emerging evidence.

In conclusion, this study demonstrates that participatory, systems-oriented strategic planning can generate actionable and context-specific pathways for strengthening climate resilience and environmental sustainability within health systems. While Iran’s health system faces substantial challenges, the findings indicate that targeted, evidence-informed, and collaborative strategies can enhance preparedness and protect population health in the face of escalating climate threats. The lessons from this study may also inform other middle-income countries seeking to operationalize climate-resilient health system frameworks under complex economic and environmental constraints.

This study has several limitations. Although community representatives and patients were not directly included in the national strategic planning committee, their perspectives, together with those of healthcare providers, were incorporated through stakeholder consultations and supporting evidence used during the planning process. In addition, while the strategic plan was informed by multiple sources of evidence, including performance indicators, literature reviews, and expert consultations, the prioritization of actions involved contextual judgment and consensus-building. Finally, the findings were developed within the specific context of Iran’s health system and may require adaptation before application in other settings. Future research should focus on evaluating the implementation of the strategic plan, assessing its impact on health system performance under climate stress, and developing monitoring frameworks for climate resilience indicators. In addition, comparative studies across similar middle-income countries are recommended to refine and validate climate-resilient health system strategies.
